# Study on evaluation method for driving fragment ability of explosives

**DOI:** 10.1038/s41598-023-40230-5

**Published:** 2023-08-10

**Authors:** Jiajie Zhou, Deren Kong, Fei Shang

**Affiliations:** School of Mechanical Engineering, Nanjing University of Science and Technology, Nanjing, 210094 China

**Keywords:** Chemistry, Engineering

## Abstract

The present study proposes an evaluation method for the driving fragment ability of explosives, which aims to provide theoretical and technical support for the selection of explosives used in warheads. The evaluation method is proposed in the light of the dimensional method and the similarity principle, and it uses TNT equivalent as the evaluation indicator. To acquire the evaluation indicator, a test system for driving fragment ability of explosives is constructed, which includes a dynamite gun type driven device, a spherical fragment, and a multi-zone fragment velocity measurement system. TNT and thermobaric explosive were used to carry out the verification experiments of the evaluation method. On the basis of the evaluation method, the basic evaluation model for the driving fragment ability of explosives was established by the TNT mass and the corresponding fragment maximum velocity. Using the basic evaluation model, the TNT equivalent of the thermobaric explosive in driving fragment ability was calculated to be 1.29, which was 3.2% different from the ratio (1.25) of both explosives’ Gurney-specific energy. The relative error of 3.2% falls within the allowable range of engineering error, confirming the feasibility of the proposed evaluation method. The result shows that the proposed evaluation method is effective and accurate in evaluating the driving fragment ability of explosives.

## Introduction

The anti-personnel warhead uses fragment as the primary destructive element. The killing ability of the fragment depends on initial parameters such as initial velocity, mass, and scatter direction. Among them, the fragment initial velocity is closely linked to the explosive driving fragment ability^1^. The explosive driving fragment ability is defined as the combined acceleration ability of the detonation waves and detonation products on the fragment. The stronger the explosive driving fragment ability, the greater the fragment initial velocity, resulting in a higher killing ability of the fragment. Therefore, to select the most suitable explosive for the anti-personnel warhead, it is very important to study evaluation method for the driving fragment ability of explosives^2–4^.

The key to accurately evaluate the driving fragment ability of explosives lies in developing a reasonable and feasible evaluation method. The feasibility of such a method depends on the evaluation indicator and the indicator acquisition method. Currently, various indicators are used to evaluate the driving fragment ability of explosives, including detonation parameters such as detonation velocity and detonation heat, Gurney energy, driven pressure index, and driven energy index. Detonation velocity, detonation heat, and other detonation parameters are typically obtained through theoretical calculations or related tests. Such indicators provide an estimate of the fragment initial velocity, which can be used to characterize the driving fragment ability of explosives. However, when comparing the estimated results for single-compound explosives and mixed explosives, that for mixed explosives tend to be more inaccurate. The Gurney energy *E*, on the other hand, refers to the total kinetic energy of the detonation products and metal, which is converted from all the chemical energy of the explosive^5^. Gurney-specific energy $\sqrt{2E}$ is a test constant that represents the performance of the explosive, and its unit is the same as that of velocity. When the charge loading ratio is in the range of 0.06 to 5.6, the Gurney-specific energy can accurately evaluate the driving fragment ability of most explosives^6–8^. The Gurney-specific energy is typically obtained by using the standard cylinder test^9^, but this method is not suitable for all kinds of explosives. For example, the high explosives can cause the shell to deform and crush rapidly, resulting in premature leakage of explosion gas products and significantly shortening the expansion loading process of the shell. Obtaining accurate Gurney-specific energy through existing measurement methods in such cases is difficult. The driven pressure index and driven energy index are obtained by driving the multiparty exponential state equation or JWL state equation^10^. However, these indicators are only applicable to the evaluation of ideal explosives and not non-ideal explosives such as those containing aluminum explosives. In summary, the evaluation indicators of the explosives driving fragment ability and the indicator acquisition methods need further study.

To enhance the universality of the evaluation indicator and simplify the indicator acquisition method, an evaluation method for driving fragment ability of explosives is proposed. The method uses TNT equivalent as the evaluation indicator. In addition, a test system for driving fragment ability of explosives is constructed. Relevant experiments were conducted to verify the feasibility of the evaluation method.

## Evaluation method for driving fragment ability of explosives

The fragment maximum velocity is defined as the fragment initial velocity. The fragment maximum velocity, which is the characterization parameter of explosives driving fragment ability, depends on several factors such as detonation parameters, shell materials, parameters of pressure transfer gas, fragment parameters, and detonation mode. After the explosive detonates, the resulting detonation waves and detonation products act on the fragment and accelerate it to the maximum velocity (*v*_0_(m/s)) in a very short time. Among the factors affecting the driving fragment ability of explosives, independent physical quantities related to the cylindrical charge include explosive density (*ρ*_*e*_(g/cm^3^)), explosive diameter (*d*_*e*_(cm)), explosive mass (*m*_*e*_(g)), detonation velocity (*D*_*e*_(m/s)), and explosive detonation multiparty index (*γ*). The independent physical quantity related to the shell is the shell density (*ρ*_*k*_(g/cm^3^)). The independent physical quantities related to the pressure transfer gas include gas density (*ρ*_*q*_(g/cm^3^)), gas volume (*V*(cm^3^)), and specific heat (*C*_v_) of the gas at constant volume. The independent physical quantities of the fragment include fragment mass (*m*_*d*_(g)) and fragment force area (*s*(cm^2^)). In summary, the fragment maximum velocity (*v*_0_) is related to the above eleven independent physical quantities:

1 $$ v_{0} = f(\rho_{e} ,d_{e} ,m_{e} ,D_{e} ,\gamma ,\rho_{k} ,\rho_{q} ,V,C_{V} ,m_{d} ,s) $$

Taking *m*_*e*_, *D*_*e*_ and *V* as basic quantities, the Eq. (1) can be transformed into the Eq. (2) using the dimension analysis:

2 $$ \frac{{v_{0} }}{{D_{e} }} = f\left( {\frac{{\rho_{e} V}}{{m_{e} }},\frac{{d_{e} }}{{V^{1/3} }},\gamma ,\frac{{\rho_{k} V}}{{m_{e} }},\frac{{\rho_{q} V}}{{m_{e} }},C_{V} ,\frac{{m_{d} }}{{m_{e} }},\frac{s}{{V^{2/3} }}} \right) $$

If the explosive type, explosive diameter, detonation mode, fragment parameters, shell material, and type of pressure transfer gas are the same, then:

$$ (\rho_{e} ,d_{e} ,D_{e} ,\gamma ,\rho_{k} ,\rho_{q} ,V,C_{V} ,m_{d} ,s) = const $$

Then the Eq. (2) can be simplified to the dimensionless Eq. (3):

3 $$ \frac{{v_{0} }}{{D_{e} }} = f\left( {\frac{{\rho_{e} V}}{{m_{e} }},\frac{{\rho_{k} V}}{{m_{e} }},\frac{{\rho_{q} V}}{{m_{e} }},\frac{{m_{d} }}{{m_{e} }}} \right) $$

where, *D*_*e*_, *ρ*_*e*_, *ρ*_*k*_, *ρ*_*q*_, *V* and *m*_*d*_ are all dimensioned constants. When all the variables in the equation are expressed in preset units, Eq. (3) can be transformed into a functional relationship between dimensionless quantities:

4 $$ v_{0} = f\left( {\frac{1}{{m_{e} }}} \right) = g(m_{e} ) = \alpha \cdot m_{e}^{\beta } $$

where, *α*, and *β* are dimensionless constants.

Different explosives have similar effects in driving fragments. This similarity principle implies that the functional relationship between explosive mass and fragment maximum velocity is comparable for different explosives. TNT is the most common standard for measuring explosive relative strength. As such, the driving fragment ability of TNT is taken as the evaluation basis.

The corresponding evaluation method involves determining the driving fragment ability of TNT, which involves obtaining the function equation *v*_0_ = *g*(*m*_*e*_) that relates the fragment maximum velocity to the TNT mass. This serves as the fundamental evaluation model for the driving fragment ability of explosives. Using this model and the corresponding fragment maximum velocity, the equivalent TNT mass of the explosive to be evaluated is calculated using the Newton’s iterative method. Finally, in terms of driving fragment ability, the TNT equivalent of the explosive to be evaluated is:

5 $$ k = \frac{{m_{TNT} }}{m} $$

where, *k* is the TNT equivalence; *m*_TNT_ is the equivalent TNT mass of explosives to be evaluated, g; *m* is the explosive mass, g.

## The test system for driving fragment ability of explosives

### System composition and principle

**Figure 1:**
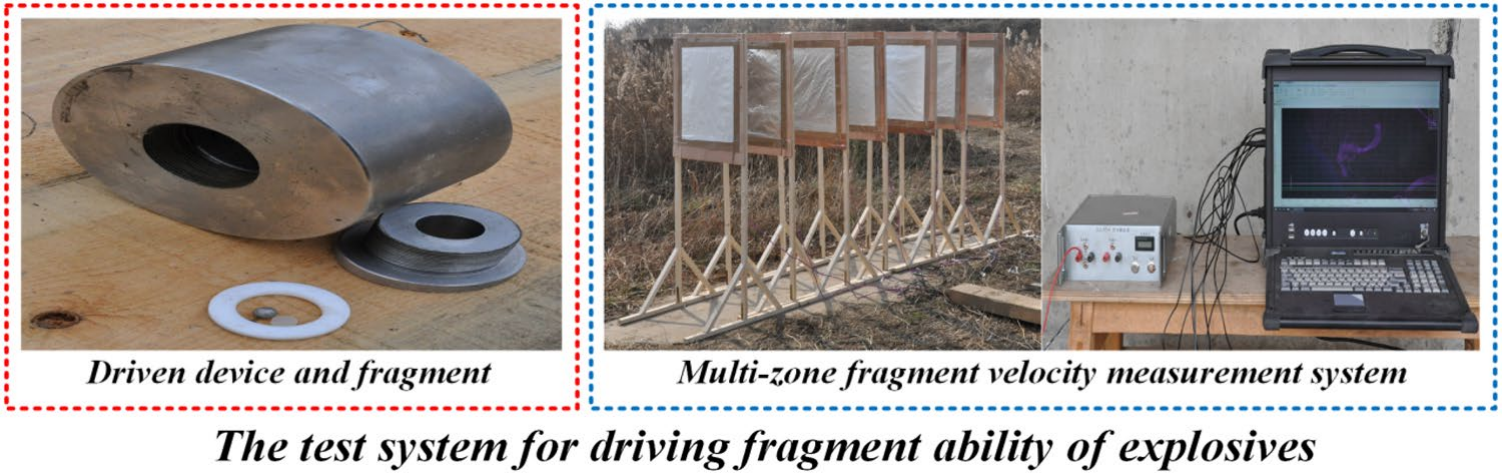
Schematic diagram of test system composition.

The purpose of constructing the test system is to determine the TNT equivalent of the explosive in driving fragment ability. Figure 1 illustrates the test system, which consists of three main components: the driven device^11^, the fragment, and the velocity measurement system. The test system operates as follows:

Detonation of the explosive, including TNT and the explosive being evaluated, is initiated in the charging chamber of the driven device, causing the detonation waves and products to propel the fragment placed in the firing chamber of the driven device. The fragment velocity at multiple measurement points is measured using the multi-zone fragment velocity measurement system. Using this data, the fragment velocity decay model can be established, allowing for the determination of the fragment maximum velocity. The obtained fragment maximum velocity and the corresponding TNT mass are used to establish the basic evaluation model for driving fragment ability of explosives. Finally, the TNT equivalent of the evaluated explosive can be determined through the basic evaluation model and the corresponding fragment maximum velocity.

In order to eliminate the impact of changes in the windward area of the fragment on the fragment velocity decay law, a tungsten alloy with high yield strength and minimal deformation is selected as the fragment material, and a sphere is used as the fragment shape. Experimental conditions such as atmospheric pressure, temperature, and humidity can have an effect on the accuracy of obtaining the evaluation indicator. Hence, in order to enhance the reliability of the evaluation result, it is crucial to maintain consistency in the driven device, fragment, and velocity measurement system, and to ensure that the experiment conditions remain as constant as possible.

### Design and simulation analysis of driven device

#### Design scheme

**Figure 2:**
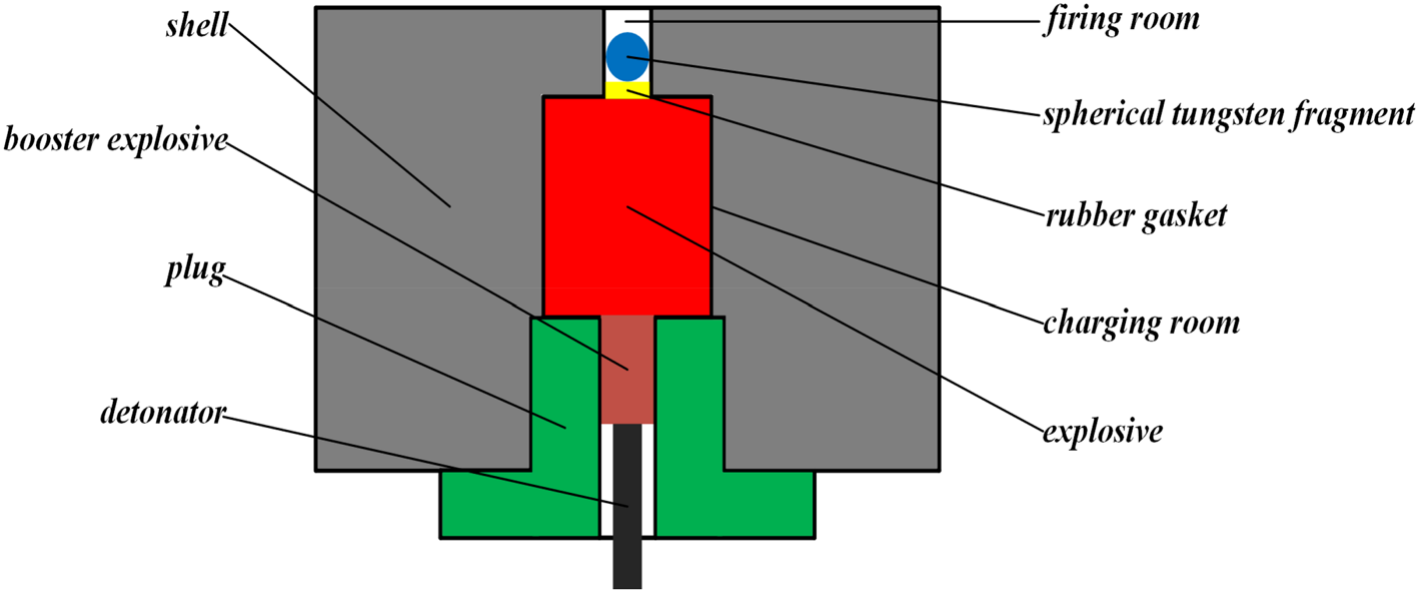
Assembly diagram of the driven device.

To reduce the number of fragments passing through the target, a dynamite gun type driven device that drives only a single fragment, is used. The high temperature and high-pressure gas formed by detonating the explosive, are used to launch the fragment by the driven device. The driven device consists of a shell, charging chamber, and firing chamber. To optimize the testing period and cost, the overall structure of the driven device is designed with a small size. Figure 2 shows the assembly schematic of the driven device.

In the design of the shell, the primary focus is on selecting the appropriate material to ensure that it remains intact until the fragment exits the firing chamber. The solid surface pressure and density exhibit a positive correlation as expressed by the relationship ∂*p*/∂*m*_0_ > 0. This indicates that, with the same shell structure design, the higher the density of the shell material, the greater the pressure on the inner wall of the shell. However, this also increases the likelihood of the shell being destroyed, and energy leakage after detonating explosive is greater, resulting in a smaller fragment maximum velocity. On the other hand, if a low-density material is used, it may lead to an incomplete detonation phenomenon, resulting in the same smaller fragment maximum velocity. Therefore, 45# steel, with a moderate density and high yield strength, is chosen as the shell material to strike a balance between solid surface pressure and solid surface density.

The charging chamber is a crucial component of the driven device, as it plays a significant role in determining the driving fragment ability of explosives. To eliminate the influence of explosive structure and diameter on the driving fragment ability of explosives, the internal structure of the charging chamber is designed to limit the explosive structure. Additionally, the explosive diameter is kept consistent with the diameter of the charging chamber. In this case, the mass of the explosive is determined by its length. However, if the explosive length is shorter than the length of the charging chamber, one end of the explosive will be directly affected by the thinning effect of air, leading to a smaller fragment maximum velocity. Therefore, a moving plug is used to adjust the length of the charging chamber, ensuring that it is consistent with the explosive length. This approach allows for the full utilization of the explosive energy and maximizes the spread of the detonation waves towards the firing chamber direction. To facilitate the installation and arrangement of the booster explosive and detonator, a space with the same diameter as the booster explosive is left in the center of the plug. Finally, single-end point detonation is used as the detonation mode, allowing for the efficient use of explosive energy.

The firing chamber plays a crucial role in accelerating the fragment in the driven device. Since the acceleration distance of the fragment is limited, the length of the firing chamber should not be too long. To control the direction of fragment movement as accurately as possible, the axis of the firing chamber should overlap with that of the charging chamber, and the detonation center should be located on the axis. Moreover, the diameter of the firing chamber and the fragment should be kept the same, and the fragment should be propelled by a bottom push. One side of the bottom push is in direct contact with the explosive, while the other side is in direct contact with the fragment. To provide the necessary elasticity, the bottom push is made of neoprene.

#### Finite element simulation model

(1) Simulation model construction.

A finite element simulation was conducted on the driven device, with parameters for the TNT explosive including a density of 1.59 g/cm^3^, diameter of 40 mm, and mass of 60 g. The fragment had a density of 17.0 g/cm^3^ and diameter of 7 mm.

**Figure 3:**
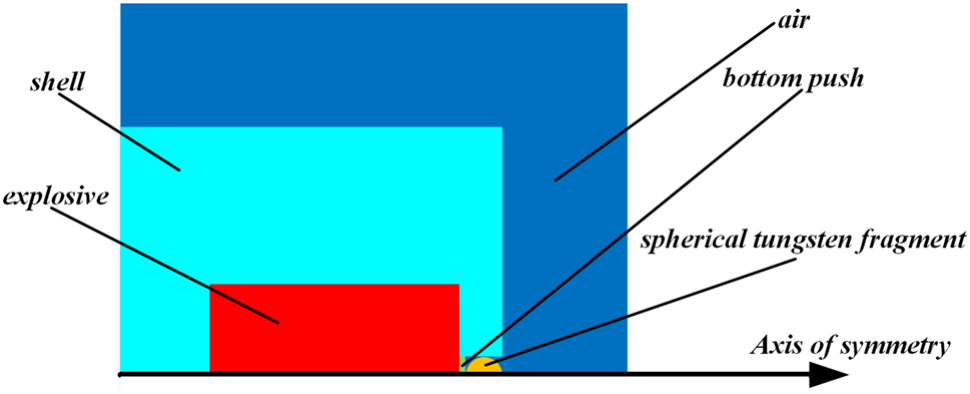
The simulation model.

The device was modeled as an axisymmetric structure, and a 1/2 2D axisymmetric model was employed for simulation to improve efficiency. The model included air, explosive, shell, bottom push (with diameter of 7 mm and thickness of 3 mm), and spherical fragment. The space for the booster explosive and detonator, which had small diameters, was filled with 45# steel. The Euler grid was used for air and TNT, while the Lagrange grid was used for the shell, bottom push, and fragment. The sizes of Euler mesh and Lagrange mesh were respectively set as 0.25 mm × 0.25 mm and 0.5 mm × 0.5 mm to ensure simulation accuracy. The fluid–solid interaction mode was Euler/Lagrange. The geometric failure strain for the shell and bottom push were both set to 2. The detonation point was located in the end face center of the charging chamber near the moving plug. The established simulation model is depicted in Fig. 3.

(2) Material model and parameters.

The ideal gas state equation and the unbiased stress dynamic model are used to describe the air. The explosive is described by the high-to-explosive burn constitutive model and the JWL equation of state, which is written as follows:

6 $$ P = A\left( {1 - \frac{w}{{R_{1} V}}} \right)e^{{ - R_{1} V}} + B\left( {1 - \frac{w}{{R_{2} V}}} \right)e^{{ - R_{2} V}} + \frac{w}{V}E $$

where, *P* is pressure; *V* is the volume; *E* is internal energy; *A*, *B, R*_1_, *R*_2_ and *w* are constants characterizing explosive characteristics. The parameter values can be found in Table 1.

**Table 1:** The CJ conditional parameters of TNT and the parameters of the JWL state equation.

Explosive	*ρ*/g·cm^−3^	*D*/m·s^−1^	*A*/GPa	*B*/GPa	*R*_1_	*R*_2_	*w*	*E*/kJ·m^−3^
TNT	1.59	6930	371.2	3.23	4.15	0.95	0.30	6.62 × 10^6^

The materials of the fragment and shell are described by the Johnson–Cook material model and Grüneisen equation of state^12^, and the material parameters can be found in Table 2. The fragment, shell, and bottom push are described by the shock equation of state, with the state equation parameters listed in Table 3.

**Table 2:** The material parameters of fragment and shell.

Material	*ρ*/g·cm^−3^	*E*/GPa	*A*/MPa	*B*/MPa	*C*	*m*	*n*	*T*_m_/K	*T*_r_/K
Tung. Alloy	17.0	344.7	1506	177	0.016	1	0.12	1723	294
45#steel	7.75	210	507	320	0.28	1.06	0.064	1765	298

**Table 3:** The state equation parameters of bottom push, fragment and shell.

Material	*ρ*/g·cm^−3^	*C*_1_/m·s^−1^	*C*_2_/m·s^−1^	*S*_1_	*S*_*2*_
Neoprene	1.439	2785	0	1.42	0
Tung. Alloy	17.0	4029	0	1.237	0
45#steel	7.75	4569	0	1.49	0

#### Analysis of simulation results

**Figure 4:**
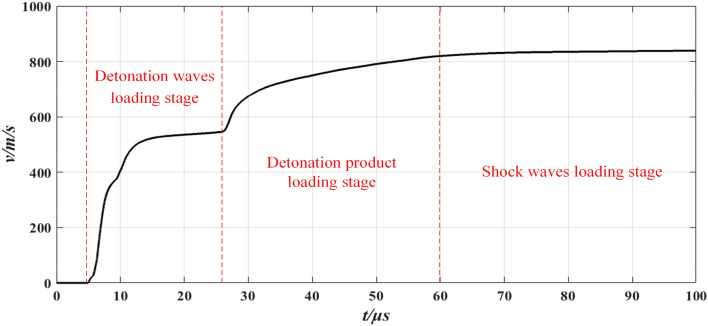
Variation curve of fragment velocity with time under 60 g TNT.

**Figure 5:**
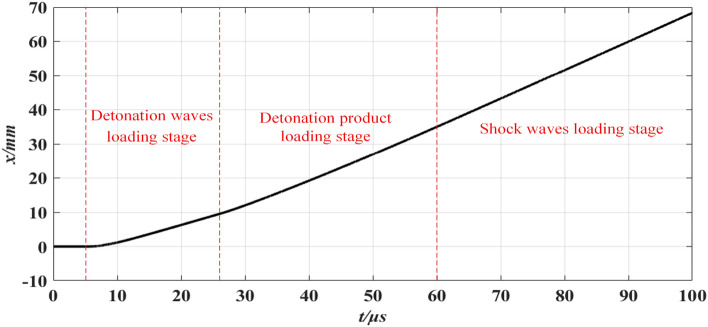
Variation curve of fragment flight distance with time under 60 g TNT.

The simulation results are presented in Figs. 4 and 5, and the subsequent analysis demonstrates that:

1. The simulation results indicate that 100 μs after detonation, the fragment velocity reached a maximum value of 839 m/s, corresponding to a flight distance of 6.83 cm. Based on these results and velocity curve, it can be concluded that the driven device is theoretically feasible.
2. As shown in Fig. 4, the process of explosive driving the fragment can be divided into three stages. The first stage is the detonation wave loading stage, which has the shortest duration but contributes the most to the acceleration of the fragment. The second stage is the detonation product loading stage, with a longer loading time of several tens of microseconds. This stage contributes the second most to the acceleration of the fragment. The third stage is the shock wave loading stage, which has the longest duration among the three stages but corresponds to the least acceleration contribution. This stage can be neglected when compared with the other two stages. From the aforementioned three stages, it can be inferred that the fragment velocity driven by the explosive is a process quantity, and only the final maximum velocity reached can reflect the driving fragment ability of explosives.
3. From Fig. 5, the corresponding position of the fragment maximum velocity is observed to be near the launch port of the driven device. However, the position obtained through simulation is only an estimate, and the fragment maximum velocity cannot be directly measured using the double-zone velocity measurement method. To deduce the fragment maximum velocity, the multi-zone velocity measurement method utilizes the fragment velocity decay model. Considering that the position where the fragment reaches the maximum velocity is very close to the launching port, the launching port can be used as the initial displacement point of the velocity decay model. Therefore, the fragment maximum velocity can be obtained through multi-zone velocity measurement.

### The construction of the multi-zone velocity measurement system

The multi-zone fragment velocity measurement system is an important tool for accurately measuring the velocity of the fragment passing through a target. The system consists of three main components, namely, a zone intercept device, a data acquisition system, and a high-precision synchronous trigger. The zone intercept device is responsible for generating a signal when a fragment passes through the target. The data acquisition system is responsible for collecting the signal generated by the fragment passing through the target and the external trigger signal. The high-precision synchronous trigger generates an external trigger signal that characterizes the time when the fragment reaches the launching port. To improve the accuracy of the velocity decay model, it is important to enhance the measurement accuracy of both the flight time and the flight distance. To accurately obtain the fragment flight distance, an aluminum foil target is used due to its ability to identify the shape of the fragment and its low cost. However, the number of aluminum foil targets used should be carefully considered to balance accurately measuring the flight distance while minimizing the risk of fragments missing the later targets. In this system, the number of aluminum foil targets is set to seven. To reduce the time measurement error as much as possible, a larger sampling rate is utilized within the permissible range of conditions. On the other hand, a broken line trigger mode is adopted in this system, where the trigger thin line is placed at the launching port. When the fragment reaches the launching port, the trigger line is broken, providing accurate measurement of the time when the fragment reaches the launching port.

**Figure 6:**
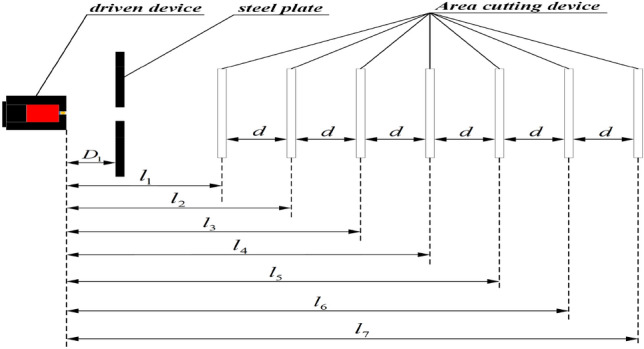
Layout scheme.

The layout scheme for the measurement system is illustrated in Fig. 6. The purpose of the steel plate is to minimize the number of natural fragments. The distance from the steel plate to the launch port of the driven device is represented by *D*_1_. The distances from each target to the launch port of the driven device are denoted by *l*_1_ through *l*_7_ indicates the distance from each target to the launch port of the driven device. The distance between each target is indicated by *d*.

## Experiment and evaluation model

### Experiment plan and measurement results

#### Experiment plan

To validate the feasibility of the evaluation method, driving fragment experiments were conducted on explosives ranging from 60 to 140gTNT and 80, 100 g Thermobaric explosive (TBE) using the test system. The measurement results were used to test the effectiveness of the driven device and establish the fundamental evaluation model for the driving fragment ability of explosives. The model was utilized to obtain the TNT equivalent of TBE in terms of driving fragment ability. The TNT equivalent and Gurney-specific energy ratio of both explosives were compared to verify the evaluation method's feasibility.

To eliminate the effect of explosive diameter, both explosives were manufactured with a diameter of 40 mm. The TBE utilized in the experiments was comprised primarily of HMX, aluminum powder, oxidizer, and adhesive, with a corresponding density of 1.88 g/cm^3^ and a detonation velocity of 9150 m/s. JH14 was chosen as the booster explosive and positioned at the end face center of the charging chamber near the moving plug. To confirm the single-shot experiment's reliability, an additional quantity of 100 g TNT and 100 g TBE were added separately. The experimental samples are listed in Table 4.

**Table 4:** Explosive samples.

Explosive type	Mass/g	Quantity
TNT	60	1
	80	1
	100	2
	120	1
	140	1
TBE	80	1
	100	2

**Figure 7:**
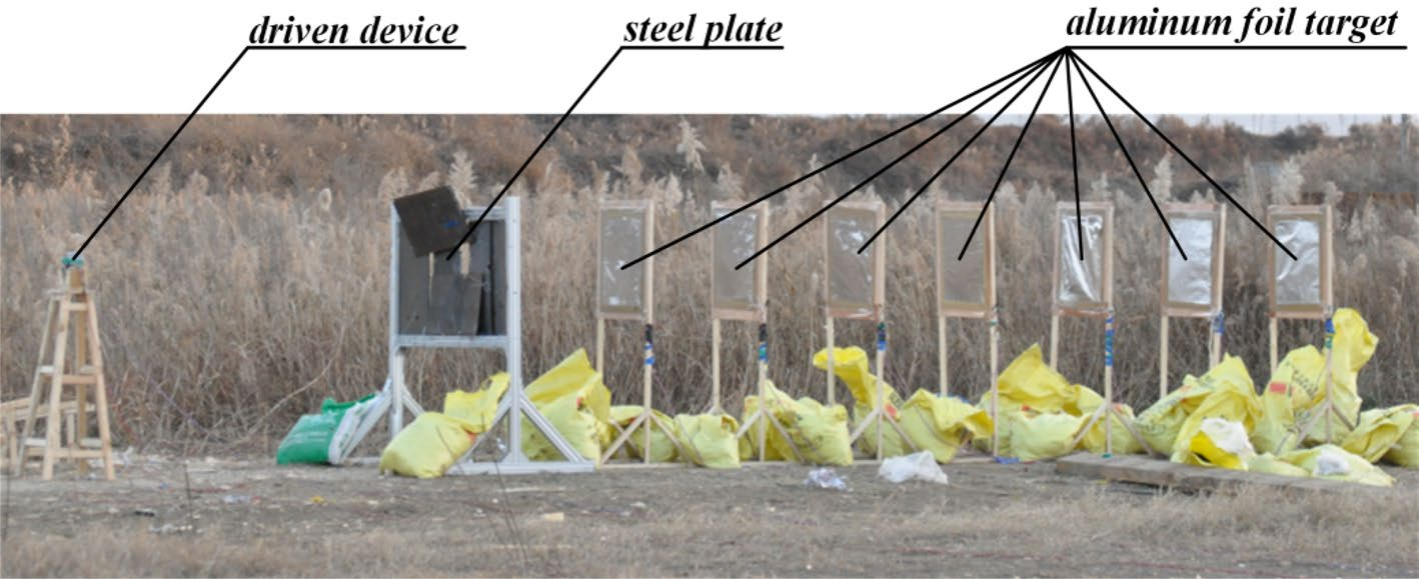
Experiment site layout.

The experiment site layout is illustrated in Fig. 7. The driven device was placed on the bomb rack, and the corresponding height of the detonation center was set to 1 m. The first aluminum foil target was placed at a distance of 3.5 m from the detonation center. To ensure that the fragments hit the targets and not miss them, each target surface was perpendicular to the ground, and the center height of each target was set to the same height as the detonation center. In order to capture the complete signal, a sampling rate of 1 MHz was selected.

#### Measurement results

After detonation, the fragment is accelerated by the detonation products and waves. Once the fragments reach their maximum velocity, they predominantly undergo straight deceleration due to air resistance. Thus, by measuring flight time (*t*) and distance (*L*) using a multi-zone fragment velocity measurement system, the velocity decay model for the fragment can be obtained.

**Figure 8:**
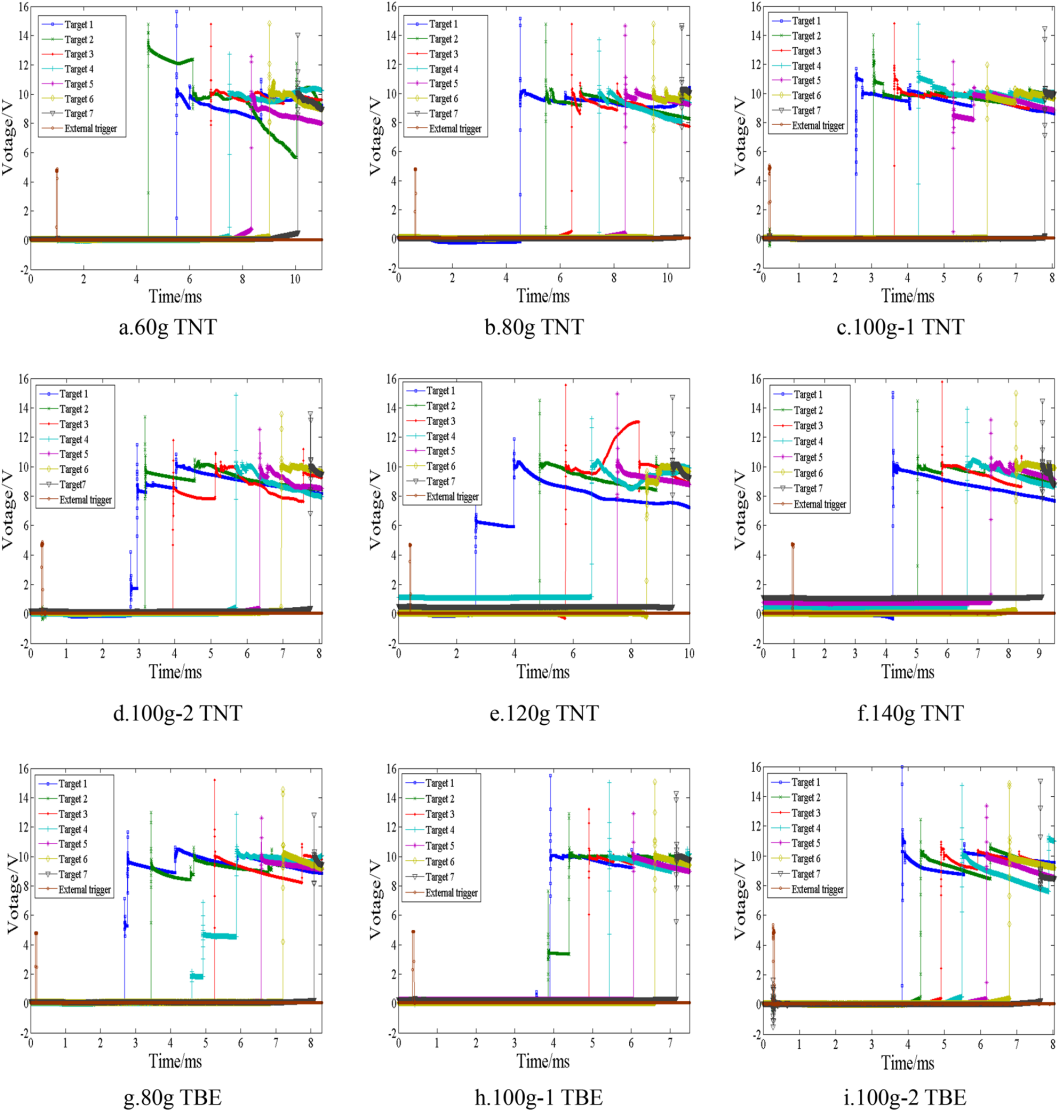
The external trigger signal and the signal generated by the fragment passing through the target.

According to the configuration of the measurement system, the fragment flight time (*t*) should be the time interval between the external trigger signal and the signal generated by the fragment passing through the target. Figure 8 displays the signal generated by the fragment passing through the target and the external trigger signal. As illustrated in the figure, the number of signals generated by the fragment passing through the target decreases as the distance between the target and the detonation center increases. This is because the driven device produces only a limited number of natural fragments after the explosive detonation, and these fragments may reach the target at a large oblique angle. Consequently, not all natural fragments that reach a previous target may necessarily reach the subsequent target. In other words, there were nearly no natural fragments that reached all targets. To accurately identify the signal generated by a spherical fragment passing through the target, it is recommended to utilize the signal of the first target as a reference. Subsequently, the correlation coefficient can be calculated to determine whether other targets exhibit signals with a high degree of similarity to the reference signal. If other targets display signals that resemble the reference signal, these signals can be identified as being generated by the fragment passing through the target. The correlation coefficient is a statistical measure that reflects the degree of linear similarity. The calculation of the correlation coefficient involves the following formula:

7 $$ \rho = \frac{{\sum {[(X_{1} - E(X_{1} ))(X_{2} - E(X_{2} ))]} }}{{\sqrt {\sum {(X_{1} - E(X_{1} ))^{2} } } \cdot \sqrt {\sum {(X_{2} - E(X_{2} ))^{2} } } }} $$

where, *X*_1_ and *X*_2_ are signals of two targets. The greater the absolute value of the correlation coefficient (*ρ*), the higher degree of similarity of the two signals.

To measure the fragment flight distance (*L*), the possible fragment holes on each aluminum foil target are screened out based on the sphere fragment shape. The fragment hole of the last target is then selected as the starting point, and the fragment holes of the other targets are identified based on their linear trajectory. Subsequently, a high-precision laser range finder is utilized to measure the distance between the detonation center and the fragment hole, representing the flight distance (*L*). The flight time (*t*) and flight distance (*L*) obtained through the above methods are presented in Table 5.

**Table 5:** The data sheet of the time (*t*) and the distance (*L*) when the fragment flies to the target.

Charge	Target sequence	Flight distance/mm	Flight time/ms	Charge	Target sequence	Flight distance/mm	Flight time/ms	Charge	Target sequence	Flight distance/mm	Flight time/ms
60 g TNT	I	3620	4.546	80 g TNT	I	3500	3.904	100 g-1 TNT	I	3594	3.856
	II	4100	5.171		II	4330	4.861		II	4075	4.385
	III	4605	5.840		III	5150	5.821		III	4560	4.928
	IV	5130	6.547		IV	6010	6.843		IV	5090	5.53
	V	5731	7.368		V	6812	7.809		V	5685	6.215
	VI	6230	8.057		VI	7670	8.854		VI	6195	6.810
	VII	6995	9.122		VII	8525	9.907		VII	6895	7.637
100 g-2 TNT	I	3536	3.734	120 g TNT	I	3500	3.651	140 g TNT	I	3500	3.292
	II	4005	4.250		II	4360	4.555		II	4360	4.092
	III	4516	4.820		III	5190	5.455		III	5180	4.891
	IV	5020	5.390		IV	5990	6.344		IV	5990	5.708
	V	5603	6.055		V	6753	7.212		V	6740	6.487
	VI	6120	6.649		VI	7560	8.144		VI	7505	7.303
	VII	6820	7.458		VII	8320	9.033		VII	8280	8.159
80 g TBE	I	3620	3.993	100 g-1 TBE	I	3600	3.546	100 g-2 TBE	I	3600	3.565
	II	4090	4.527		II	4080	4.031		II	4070	4.080
	III	4600	5.117		III	4580	4.545		III	4580	4.635
	IV	5138	5.745		IV	5082	5.068		IV	5085	5.215
	V	5738	6.450		V	5673	5.691		V	5660	5.899
	VI	6260	7.067		VI	6190	6.240		VI	6175	6.526
	VII	7012	7.957		VII	6710	6.796		VII	6855	7.386

### Method of obtaining the fragment maximum velocity

Due to the small weight of the fragments used in the experiment, the effect of gravity can be ignored, and it can be assumed that the fragment motion is solely affected by air resistance. Accordingly, the motion equation of the fragment can be derived using Newton's second law:

8 $$ m\frac{dv}{{dt}} = - \frac{1}{2}\rho Scv^{2} $$

In the equation, *m* represents the mass of the fragment; *ρ* denotes the air density; *S* represents the windward area of the fragment. For spherical fragments, the windward area is given by S = π*r*^2^, where *r* is the radius of the fragment. *c* is the air resistance coefficient; *v* represents the velocity of the fragment; *t* denotes the flight time of the fragment. By rearrange Eq. (8) and integrating both sides, we can obtain:

9 $$ \int {\frac{dv}{{v^{2} }} = - \frac{c\rho S}{{2m}}\int {dt} } $$

With the initial condition *t* = 0 and *v* = *v*_0_, the integral equation can be solved as follows:

10 $$ v = \frac{{2mv_{0} }}{{c\rho Sv_{0} t + 2m}} $$

And *v* = *dL*/*dt* = *dL*·c*ρSv*_0_/*d*(2* m* + c*ρ*S*v*_0_*t*), let *k* = c*ρS*, then:

11 $$ \frac{k}{2m}dL = \frac{{d(2m + kv_{0} t)}}{{2m + kv_{0} t}} $$

With the initial condition *t* = 0 and *L* = 0, the differential equation can be solved as follows:

12 $$ t = \frac{2m}{{kv_{0} }}(e^{{\frac{k}{2m}L}} - 1) $$

The exponential part of Eq. (12) is expanded by Taylor:

13 $$ t = \frac{2m}{{kv_{0} }}\sum\limits_{n = 1}^{\infty } {\frac{1}{n!} \cdot (\frac{kL}{{2m}})^{n} } $$

According to Eq. (13), the relationship between the fragment flight time (*t*) and distance (*L*) can be represented by a polynomial function. Hence, the method of least squares can be employed to perform polynomial fitting of the (*L*_*i*_, *t*_*i*_) data points:

14 $$ t = a_{0} + a_{1} L + \cdots + a_{m} L^{m} $$

where, *a*_0_ ~ *a*_m_ is the fitting coefficient.

When applying the least squares method for polynomial fitting, it can be challenging to determine the appropriate degree of the polynomial. To ensure that the sum of squared residual errors associated with the chosen degree of the polynomial is minimized, the F-test is utilized.

After determining the degree M of the polynomial through F-test, both sides of Eq. (14) are differentiated with respect to *t* to obtain the relationship between flight distance and velocity. Therefore, the model for the decay of fragment velocity can be expressed as:

15 $$ v(L) = \frac{1}{{a_{1} + 2a_{2} L + \cdots + a_{M} L^{M} }} $$

where, *v* (*L*) is the fragment velocity corresponding to the flight distance *L*. When *L* is 0, the fragment maximum velocity is given by *v*_0_ = 1/ *a*_1_.

**Figure 9:**
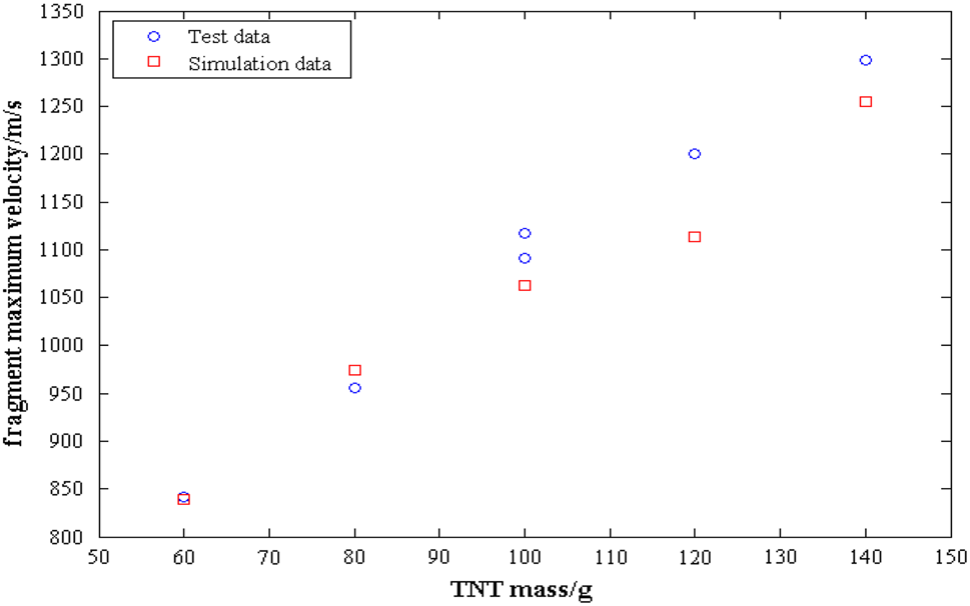
The comparison diagram between test data and simulation data.

The aforementioned methods are applied to process the data presented in Table 5, and the resulting calculation outcomes are displayed in Table 6. Table 6 also presents the simulation data concerning TNT. The comparison between the test and simulated results for the fragment maximum velocity is illustrated in Fig. 9. Based on the analysis of Table 6 and Fig. 9, the following conclusions can be drawn:

1. The fragment maximum velocities resulting from two rounds of 100 g TNT or two rounds of 100 g TBE are similar, indicating that the results of the single shot experiment are reliable and the driven device is feasible.
2. The absolute relative error between the test and simulation results is below 10%, indicating that the simulation model is accurately constructed and the simulation results are reliable. The main reason for the differences between the two sets of results is that the shell in the simulation model is treated as a single unit, whereas the shell used in the experiment is a composite structure. Therefore, the simulation model struggles to accurately replicate the strength of the driven device.
3. As the explosive mass increases, the corresponding maximum fragment velocity also increase. Additionally, for the same mass of explosives, TBE produces a greater fragment maximum velocity than TNT. This suggests that there is a similar functional relationship between explosive mass and fragment maximum velocity for different explosives. Furthermore, TBE has a stronger ability to drive fragments compared to TNT when the mass of explosives is the same.

**Table 6:** The fragment maximum velocity for each explosive.

Charge	*v*_*0*_ of test*/* m·s^−1^	*v*_*0*_ of simulation m·s^−1^	Relative error/%
60 g TNT	841.75	839.00	0.33
80 g TNT	956.16	974.63	− 1.90
100 g-1 TNT	1091.40	1063.02	2.67
100 g-2 TNT	1117.05	1063.02	5.08
120 g TNT	1200.70	1113.62	7.82
140 g TNT	1298.10	1254.65	3.46
80 g TBE	1095.86	–	–
100 g-1 TBE	1267.30	–	–
100 g-2 TBE	1245.20	–	–

### Establishment and test of the evaluation model

**Figure 10:**
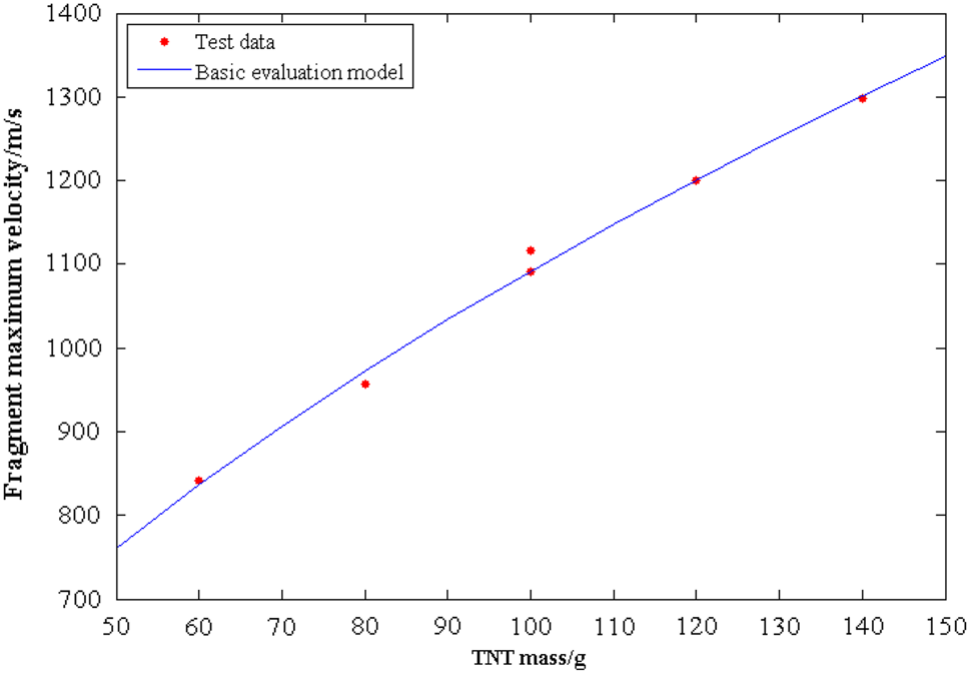
The comparison diagram between fitting results and test data.

To obtain the *g* (*m*_e_) function between the fragment maximum velocity and the TNT mass, a basic evaluation model is fitted using the least squares method based on the data in Table 6. The fitting result is:

16 $$ v_{0} = 99.02 \times m_{e}^{0.5212} $$

where, *m*_e_ represents the mass of TNT, *v*_0_ represents the fragment maximum velocity. The fitting results of the basic evaluation model compared to the measured maximum velocity are presented in Fig. 10. It can be observed from the graph that the model fitting results closely align with the measured maximum velocity, indicating that the model serves as a reliable basic evaluation model. Equation (16) is specific to the experimental conditions used in this study, including the explosive, driven device, and fragment used.

The feasibility of the evaluation method was verified by substituting the fragment maximum velocity corresponding to 80 g and 100 g TBE into the model. Through an iterative process, the equivalent TNT mass was determined as 100.72 g, 133.12 g, and 128.70 g, respectively. Utilizing Eq. (1), the TNT equivalence of TBE in terms of the driving fragment ability was determined to be 1.29. The detonation velocity of TBE and TNT was then converted into Gurney energy, revealing a ratio of TBE to TNT Gurney-specific energy of 1.25. Notably, the difference between the TNT equivalence obtained and the ratio of both explosives' Gurney-specific energy was calculated to be 3.2%. The relative error of 3.2% falls within the allowable range of engineering measurement error, confirming the feasibility of the proposed evaluation method.

TNT and TBE are categorized as ideal and non-ideal explosives, respectively. The proposed evaluation method can be utilized to assess the driving fragment abilities of both types of explosives. When evaluating the driving fragment abilities of other explosives, as long as the driven device, driven fragments, and velocity measurement system used for the explosive are consistent with the proposed evaluation method, the obtained basic evaluation model can be used to determine the TNT equivalence of other explosives in terms of driving fragment abilities. Furthermore, during the evaluation process, the density, diameter, and length of the explosive should be obtained through measurements. Explosion parameters such as detonation velocity can be obtained through theoretical calculations or measurements. The TNT equivalence of the explosive can be determined by combining measurements and calculations.

## Conclusion

In this study, an evaluation method for driving fragment ability of explosives was proposed, aimed at addressing the non-universality of the existing evaluation indicator and the complexity of the corresponding indicator acquisition method. Analyzing the proposed evaluation indicator and the constructed test system, the following conclusions are obtained:

1. By utilizing the TNT equivalent as the evaluation indicator, the proposed method offers a quantitative and objective approach to assess the driving fragment ability of explosives, regardless of their specific chemical properties or structural characteristics. This is because the TNT equivalent is a widely accepted standard for comparing the performance of explosives. Furthermore, the use of the dimensional method and similarity principle in developing the evaluation indicator ensures that the indicator is based on fundamental physical principles, which enhances the validity and reliability of the evaluation method.
2. A test system was designed using the principle of explosive gun and conventional materials, resulting in a short production cycle and low cost. The velocity measurement method used was the multi-zone intercept velocity measurement method, with a corresponding conventional target, which made the velocity measurement system easy to establish and independent of the explosive type. The test system was found to be simpler compared to other indicator acquisition methods such as the cylinder test.

In summary, the proposed evaluation method is effective and accurate in evaluating the driving fragment ability of explosives, and provides theoretical and technical support for the selection of explosives used in warheads. Future studies could explore the application of the proposed evaluation method to other types of explosives, as well as further validating the basic evaluation model established in this study.

## Data Availability

The datasets used and/or analyzed during the current study available from the corresponding author on reasonable request.

## References

[1] Tang JJ, Liang ZF, Qu KP (2020). Correlation between the mechanical properties of tungsten alloy fragments and fracture behavior driven by detonation loading. Chin. J. Explos. Propell..

[2] Li W, Huang GY, Feng SS (2015). Effect of eccentric edge initiation on fragment velocity distribution of a cylindrical casing filled with charge. Int. J. Impact Eng.

[3] Wang L, Han F, Zhou Q (2017). The projection angles of fragments from A cylindrical casing filled with charge initiated at one end. Int. J. Impact Eng.

[4] Ren GW, Guo ZL, Fan C (2016). Dynamic shear fracture of an explosively-driven metal cylindrical shell. Int. J. Impact Eng.

[5] Grisaro HY, Dancygier AN (2018). Characteristics of combined blast and fragments loading. Int. J. Impact Eng.

[6] Li X, Wang WL, Liang ZF (2022). Research progress on acceleration ability of explosive detonation to metal shell. J. Proj. Rockets Missil. Guid..

[7] Dany F (2016). Simple correlations for the estimation of propellants specific impulse and the gurney velocity of high explosives. Combust. Sci. Technol..

[8] Dany F (2015). A simple relationship for the calculation of the gurney velocity of high explosives using the BKW thermochemical code. J. Energ. Mater..

[9] Wang XY, Wang SS, Ma F (2018). Experimental study on the expansion of metal cylinders by detonation. Int. J. Impact Eng..

[10] Wang XY, Wang SS, Xu YX (2014). Conversion of explosive damage energy of high explosive warhead. Acta Armamentarii.

[11] Huneault J, Loiseau J, Hildebrand MT (2022). An explosively driven launcher capable of 10km·s^−1^ projectile velocities. Shock Waves.

[12] Chen XW, Wang JX, Tang K (2020). Analysis on the initial velocity field of a multi-layer spherical fragment driven by explosion. J. Vib. Shock.

